# Comparison of two blind brachial plexus blocks in goat cadavers

**DOI:** 10.5455/javar.2025.l872

**Published:** 2025-03-24

**Authors:** Sunisa Sirimongkolvorakul, Tanasid Sornphu

**Affiliations:** Department of Preclinical Science, Faculty of Veterinary Medicine, Mahanakorn University of Technology, Bangkok, Thailand

**Keywords:** Additional approach, brachial plexus block, goat cadaver, methylene blue, traditional approach

## Abstract

**Objective:**

This study aimed to compare two approaches to the brachial plexus: the traditional blind method and an additional technique, both of which use anatomical landmarks to guide needle insertion.

**Materials and Methods:**

The traditional and additional approaches were performed on both thoracic limbs of 24 cadavers (24 for each approach). Methylene dye is used for injection and nerve staining. We counted the nerves that successfully stained (staining less than 1 cm). The Mann–Whitney *U* test was used to compare approaches.

**Results:**

The findings indicated that all cadavers were successfully used. The traditional approach and the additional approach revealed success rates of 45.83% and 54.17%, respectively. We found no significant differences between the two techniques (*p* > 0.05).

**Conclusion:**

The additional approach presents a viable alternative to the traditional method for performing the brachial plexus block in goats. Further research into the clinical differences between these techniques could lead to useful insights that help make them more accurate and useful.

## Introduction

The brachial plexus block is widely utilized in veterinary anesthesia to provide intraoperative analgesia during forelimb surgeries. These nerve blocks prevent the transmission of nociceptive signals from peripheral sensory nerves to the central nervous system, effectively blocking pain perception [1]. Various approaches have been documented, each designed to target specific areas of the brachial plexus, depending on the surgical site requiring desensitization [2–4]. Several techniques have been described in dogs and cats for performing this block [5–7]. However, there are not many studies on how it works in goats, so it is hard to say how to do an accurate and effective brachial plexus block on these animals. Therefore, a better understanding of the brachial plexus block in goats could play a vital role in improving its use in practice as well as supporting further research and enhanced animal care.

The brachial plexus is a complex network of nerves originating from the ventral branches of the sixth (C6), seventh (C7), and eighth (C8) cervical nerves, as well as the first (T1) thoracic and second (T2) thoracic spinal nerves in caprine species, including goats. It primarily innervates the thoracic limb [2,8]. Typically, the brachial plexus consists of the suprascapular, subscapular, musculocutaneous, axillary, radial, median, and ulnar nerves, similar to most species. Among these, the radial nerve is the largest and provides motor innervation to the extensor muscles of the arm, while the median and ulnar nerves innervate the flexor muscles [8].

In canine patients, the traditional approach to the plexus is a commonly used technique for performing a brachial plexus block. Although recent advancements, nerve stimulator-assisted or ultrasound-guided blocks have been developed, the blind approach is still frequently employed because it does not require additional equipment or advanced skills [9–12]. Over the past decade, the goat population in Thailand has increased, reflecting a growing demand for goat meat and milk. To facilitate this expansion, the Thai government has introduced policies aimed at promoting goat farming, including initiatives such as the provision of free vaccinations and artificial insemination services [13].

The distribution of the goat population is predominantly concentrated in areas with substantial Thai Muslim communities, particularly in the southernmost provinces and Nong Chok, a district located on the periphery of Bangkok. Veterinary clinics frequently encounter goats presenting with musculoskeletal disorders and injuries affecting the appendicular skeleton. Nonetheless, there remains a paucity of research offering precise and evidence-based methodologies for performing brachial plexus blocks in goats. Advancing knowledge in this area holds the potential to enhance veterinary practices, support research endeavors, and improve the overall health and welfare of goat populations.

Additionally, economic constraints and the limited availability of anesthetics and analgesics for small ruminants may influence the choice of technique [3]. Inhalation anesthesia is rarely feasible or economically justified, except in cases where the animal holds significant economic value [3,14]. The identification of anatomical landmarks for the traditional approach to the brachial plexus presents considerable challenges, particularly in heavily muscled or obese goats, frequently leading to unsuccessful nerve blocks. An alternative technique involves targeting the brachial plexus nerves at their emergence proximal to the shoulder joint. The objective of this study was to compare the efficacy of the traditional blind approach to the brachial plexus in goat cadavers with an alternative method that incorporates anatomical landmarks to guide needle placement. It was hypothesized that the alternative approach would be more straightforward to execute and achieve superior dye distribution compared to the traditional method for brachial plexus blockade in goats.

## Materials and Methods

### Ethical approval

This study was conducted in the Department of Anatomy at the Faculty of Veterinary Medicine, Mahanakorn University of Technology, with approval from the Mahanakorn University of Technology Institutional Animal Care and Use Committee (ACUC-MUT-2024/003). We used a total of 24 native goats, one brachial plexus for each approach. These cadavers were sourced for the undergraduate dissection class.

In Part I of the study, a sixth-year student with no prior experience in either approach was trained by an experienced clinician to perform both traditional and additional approaches using five cadavers. The operator intentionally lacked prior experience with either approach to minimize the biasing of the results toward a more familiar technique. Part II: Bodies of goats were fixed with a 4% formaldehyde solution. Then, 24 goats were randomly assigned to one of two treatments: the traditional approach or the additional approach. A coin toss determined which approach would be performed first, as well as whether the right or the left thoracic limb would be used initially. We then used the opposite limb for the remaining approach, ensuring that each cadaver received both treatments. The cadaver was placed in lateral recumbency for both approaches; the hair was clipped bilaterally. Methylene blue dye (0.1%, pH 7.34; Sigma-Aldrich, Inc., MO, USA) was used for injection and nerve staining. The total volume of diluted dye used for each approach was equivalent to a volume of 0.1 mg/kg Xylazine [15]. Following the completion of the dye infusion, which took 5 min, we carefully dissected the designated area and observed the brachial plexus nerves with our naked eyes.

### Techniques for injection

**Figure 1.**
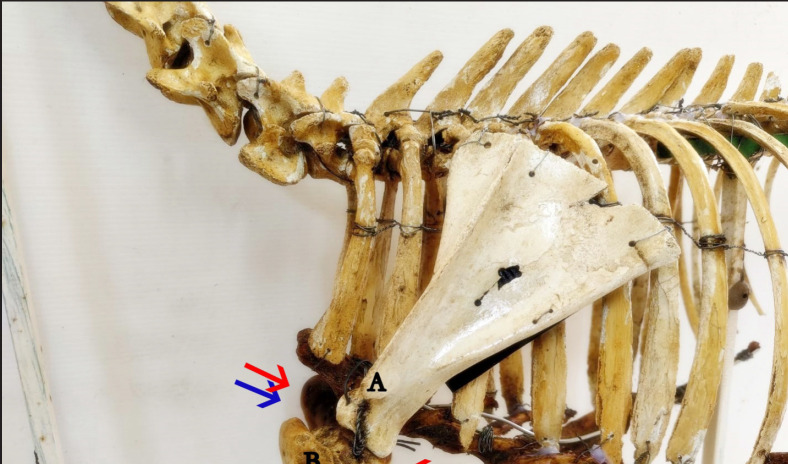
Illustration of the traditional approach (blue needle) and the additional approach (red needle) to the brachial plexus nerve block in goat cadavers. Note that in the traditional approach (blue arrow), the needle is introduced at the midpoint between the acromion process (A) and the point of the shoulder or greater tubercle of the humerus (B). In the additional approach (red arrow), the needle is introduced both cranial and caudal at the midpoint between the acromion process (A) and the point of the shoulder or greater tubercle of the humerus (B).

The traditional approach has been previously described [16]. The acromion of the scapula and the cranial border of the greater tubercle of the humerus were palpated to guide the procedure. A 23-gauge, 3.81 cm needle was then inserted parallel to the midpoint of these landmarks, medial to the median plane, to target the ventral branches of spinal nerves C6 to T2 for injection (Fig. 1). The additional approach followed the same procedure as the traditional approach for the first injection. For the second injection, the tip of the needle was put just below the shoulder joint, between the grooves of the long and lateral heads of the triceps brachii muscle. The needle was then aimed craniomedial to the joint.

### Scoring system for nerve staining

The scoring system aligns with those used in previous studies [16,17]. We identified the brachial plexus nerves (suprascapular, subscapular, musculocutaneous, axillary, radial, median, ulnar, and thoracodorsal) based on their insertions in the musculoskeletal forelimb. Successfully stained nerves (stain ≥ 1 cm) were counted. We applied the following criteria to assess dye impregnation: I—no coloration; II—weak coloration (1/4 of the nerve colored); III—medium coloration (half of the nerve colored); IV—strong coloration (3/4 of the nerve colored); and V—completely colored (nerve completely colored). We deemed the block satisfactory when the dye impregnated all nerves strongly (score IV) or totally (score V).

### Statistical analysis

The Mann–Whitney *U* test was performed to assess the difference in the number of stained nerves between approaches. We set the significance level at *p = 0.05.*

## Results

**Figure 2.**
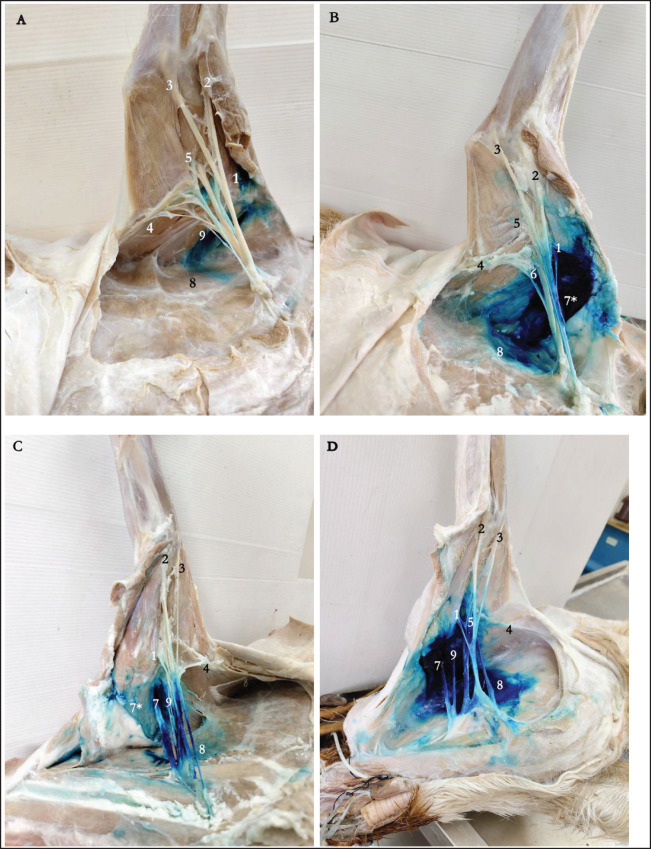
Example of brachial plexus scoring of methylene blue stain distribution after injection using two approaches for needle insertion: (a) goat 16: traditional approach, staining score I with lack of staining of any nerve with axillary injection of dye; (b) goat 5: additional approach, staining score III with dye stain of suprascapular, subscapular, musculocutaneous, and median nerves; (c) goat 8: traditional approach, staining score V with staining of all nerves; (d) goat 21: additional approach, staining score V with staining of all nerves. 1, musculocutaneous n.; 2, median n.; 3, ulnar n.; 4, thoracodorsal n.; 5, radial n.; 6, axillary n.; 7, suprascalular n.; 7*, supraspinatus m.; 8, serratus ventralis m.; 9, subscapular n.

Forty-eight brachial plexuses from 24 goat cadavers, with an average body weight of 17.44 ± 0.44 kg, were examined, with 24 plexuses allocated to each approach. There were no significant differences in success rates between the two techniques. Incomplete staining (scores I, II, and III) was present in 11 out of 24 plexuses (45.83%) using the traditional approach and in 13 out of 24 plexuses (54.17%) using the additional approach (Fig. 2b). For score I (Fig. 2a), the dye was not in contact with any nerves and was instead located in the surrounding area of the plexus, including the prescapular lymph node, supraspinatus muscle, subscapularis muscle, or triceps brachii muscle. Thirteen out of 24 plexuses (54.17%) had complete staining (score IV or V) when the traditional method was used, and 11 out of 24 plexuses (45.83%) when the extra method was used. We assigned score V to the staining of all musculocutaneous, median, ulnar, thoracodorsal, radial, axillary, suprascapular, and subscapular nerves. The additional approach (Fig. 2d) demonstrated better distribution of the dye compared to the traditional approach (Fig. 2c). No intrathoracic injections were noted. However, instances of intravascular injection into the axillary vein were observed in 2 out of 24 cases (8.33%) with the additional approach.

## Discussion

Local anesthetics possess the unique ability to block pain sensation and have long served as adjuncts to light general anesthesia in both small and large animals [18]. Regional anesthesia lowers the overall need for anesthetic, speeds up recovery, and stops the central sensitization of the pain pathway after painful surgeries, which means that less pain medication is needed afterward [19]. The brachial plexus block is a traditional technique employed for procedures involving the forelimbs. In goats, a brachial plexus block is done by making the ventral roots of the sixth (C6), seventh (C7), and eighth (C8) cervical nerves less sensitive. The first (T1) and second thoracic (T2) spinal nerves must also be desensitized [2,8]. Various techniques for performing the block have been documented in dogs and cats [5–7]. In canine patients, the traditional approach to the plexus is commonly used for administering a brachial plexus block.

A previous study [20] reported that goats and sheep with lower adult body weights might be suitable candidates for limb amputation following irreparable limb injuries. They utilized a brachial nerve block for forelimb amputations and found that the prognosis for survival and recovery from surgery was excellent. This study demonstrated that both blind techniques could effectively reach the brachial plexus, as indicated by the implantation of dye in goat carcasses. To prevent biasing results, the same individual, who had no prior experience with either approach, consistently performed the block. The findings indicated that all cadavers were successfully used. In our study, the traditional approach and the additional approach revealed success rates of 45.83% and 54.17%, respectively. The rate of complete success was higher than the rate of 30% previously reported in dog cadavers [21]. Species variation may account for this difference [22]. Moreover, another study found that nerve depths were positively correlated with the weight of animals [5].

According to our study, scores of IV and V indicated a complete block, achieved in 54.17% of plexuses using the traditional approach and 45.83% using the additional approach. There were no significant differences in success rates between the two techniques. These scoring data are consistent with recent studies in dog cadavers, which are associated with complete dying of nerves [16,21]. Additionally, previous studies have used methylene blue to evaluate successful nerve targeting, labeling nerves stained by less than 1 cm as successfully blocked. Clinical applications widely utilize methylene blue as a stain. Previous research has shown that a lidocaine solution containing methylene blue may provide an effective method for studying the spread of injectate of various nerve blocks in post-mortem studies [16,21,23]. A drug exposure distance of at least 0.5 cm is required for a successful block, but the exact distance depends on the concentration of the local anesthetic [24]. Because of differences in physics and chemistry, the dye staining method used in this study might not accurately show how the local anesthetic spread, but it is still a good way to test new regional block techniques on dead bodies. Moreover, dye distribution may vary between fresh cadavers and formaldehyde-embalmed cadavers.

Some problems that can happen with the old method are intrapleural space injection, intravascular injection, and direct nerve injury [7,25–27]. The axillary artery and vein lie medial to the root of the brachial plexus, while the musculocutaneous median ulnar trunk is caudal to the artery. The axillary vein terminates caudally to the trunk [8,28]. Our results showed an 8.33% occurrence of intravascular injection into the axillary vein with the additional approach. However, due to the limitations of this study, the use of cadavers, and the risk of vascular puncture, it could not be fully assessed. Aspiration of the needle prior to injecting local anesthetic is always recommended to ensure that the needle is not within the pleural space or a blood vessel.

To successfully block the target nerves, it is important to accurately locate anatomical landmarks and place the needle in relation to the plexus. Because of this accuracy, smaller amounts of anesthetic have been used in several studies that used electrolocation or ultrasound guidance [9,29]. Recently, Pratt and Martinez-Taboada [30] talked about a lateral approach ultrasound-guided radial, ulnar, median, and musculocutaneous nerve (RUMM) block in cat cadavers. Key landmarks identified on the ultrasound included the first rib, the greater tubercle of the humerus in the cranial near field, the RUMM nerves within their fascicle plane in the mid-field, caudal to the humerus, and surrounded by musculature and fat of the brachium.

In order to accurately administer localized anesthesia and make forelimb surgeries in goats more accurate and efficient, it is necessary to have a thorough understanding of the brachial plexus. This method works especially well for orthopedic procedures, wound healing, and other therapeutic procedures that involve the forelimb, which leads to better patient outcomes and animal welfare [31]. In clinical practice, relying on anatomical landmarks to locate the brachial plexus may be more cost-effective than techniques requiring specialized equipment. Further evaluation of this additional approach through prospective studies assessing success rates in goats undergoing surgical procedures is warranted.

## Conclusion

For performing brachial plexus blocks, the results show that both the extra approach and the traditional approach work just as well. This outcome increases the number of techniques practitioners can use, which gives them more options for getting good plexus access. To get accurate localization and improve the success rates of brachial plexus blocks, you need to know how to use anatomical landmarks. However, more research is necessary to identify any possible clinical differences between these methods.

## List of Abbreviations

ACUC, animal care and use committee; CM, centimeter; MBD, methylene blue dye; RUMM, radial, ulnar, median, musculocutaneous nerve.
